# Association between nursing assessment indicators and adverse outcomes in hospitalized patients with atrial fibrillation: A retrospective observational study

**DOI:** 10.1097/MD.0000000000051239

**Published:** 2026-10-30

**Authors:** Jie Han, Siyu Liu, Nan Li, Ruiqin Zhang

**Affiliations:** a Department of Circulation 18, Dalian University Affiliated Zhongshan Hospital, Dalian, Liaoning Province, China; b Circulatory Comprehensive Ward, Dalian University Affiliated Zhongshan Hospital, Dalian, Liaoning Province, China.

**Keywords:** Adverse outcomes, Atrial fibrillation, Fall risk, Functional status, Hospitalization, Nursing assessment, Nutritional status, Retrospective study

## Abstract

This retrospective observational study examines the association between routinely collected admission nursing assessment indicators and in-hospital adverse outcomes among hospitalized patients with atrial fibrillation (AF). A total of 312 hospitalized patients with AF admitted to a tertiary teaching hospital between January 2021 and December 2024 were included. The Morse Fall Scale, Barthel Index, Nutritional Risk Screening 2002 (NRS-2002), and Braden Scale were assessed within 24 hours of admission. Clinical characteristics, vital signs, and laboratory data were extracted from electronic medical records. The composite adverse outcome comprised in-hospital death, unplanned intensive care unit admission, worsening heart failure requiring treatment escalation, major bleeding requiring medical intervention, or thromboembolic events. Univariable and multivariable logistic regression analyses were used to examine associations with the composite outcome. Among 312 patients, 68 (21.8%) experienced at least one adverse outcome. In the adjusted analysis, a higher Morse Fall Scale score was associated with higher odds of adverse outcomes (OR 1.031, 95% CI 1.012–1.051; *P* = .002), whereas a higher Barthel Index score was associated with lower odds (OR 0.978, 95% CI 0.961–0.995; *P* = .012). A higher NRS-2002 score was associated with higher odds (OR 1.376, 95% CI 1.083–1.748; *P* = .009), and a higher serum albumin level was associated with lower odds (OR 0.953, 95% CI 0.920–0.988; *P* = .010). AF type and Braden Scale score were not independently associated with the composite outcome after adjustment. Admission nursing assessments of fall risk, functional status, and nutritional risk, together with serum albumin, were independently associated with in-hospital adverse outcomes. These results support their use as complementary markers of vulnerability and for closer clinical surveillance, but they do not establish causality or justify changing guideline-directed AF treatment. Prospective multicenter studies are required before these measures can be incorporated into validated clinical decision tools.

## 1. Introduction

Atrial fibrillation (AF) is the most common sustained cardiac arrhythmia encountered in clinical practice and is a major global public-health challenge. Its prevalence increases markedly with age and is expected to rise further as populations age. AF is associated with stroke, heart failure, mortality, recurrent hospitalization, impaired quality of life, and substantial healthcare use.^[1–4]^

Hospitalized patients with AF frequently have advanced age, multiple comorbidities, and reduced physiological reserve, which may increase susceptibility to thromboembolic events, bleeding, worsening heart failure, unplanned intensive care unit (ICU) transfer, and in-hospital death. Contemporary guidelines emphasize integrated, patient-centered AF care, dynamic reassessment, and management of comorbidities and modifiable risk factors rather than reliance on a single risk score.^[5]^

Stroke and bleeding scores such as CHA2DS2-VASc and HAS-BLED were developed for specific clinical purposes and do not comprehensively quantify functional dependence, mobility limitations, frailty-related vulnerability, or nutritional status. The 2023 ACC/AHA/ACCP/HRS guideline notes limitations of commonly used bleeding-risk scores and emphasizes individualized assessment, while the 2024 ESC guideline promotes the AF-CARE framework and repeated evaluation of the whole patient.^[5]^

Routine nursing assessments provide structured information on dimensions that are not captured directly by conventional cardiovascular risk scores. The Morse Fall Scale reflects fall-related mobility and safety risk; the Barthel Index quantifies independence in activities of daily living; NRS-2002 evaluates nutritional risk; and the Braden Scale integrates mobility, activity, nutrition, and skin-related vulnerability. These measures are noninvasive, routinely available early in hospitalization, and may identify broader physiological vulnerability.

Recent AF literature increasingly supports the prognostic relevance of frailty and malnutrition. A 2025 meta-analysis of 23 studies found frailty to be associated with higher all-cause mortality, stroke, and major bleeding in older adults with AF.^[6]^ In a prospective AF cohort, frailty was associated with major bleeding and death, and moderate-to-severe malnutrition was associated with ischemic stroke/transient ischemic attack and mortality.^[7]^ A 2026 systematic review likewise reported consistently poorer outcomes in frail older adults with AF.^[8]^ However, these studies mainly used formal frailty or nutritional instruments and were not designed to evaluate whether a set of routinely documented admission nursing scales is independently associated with short-term in-hospital deterioration.^[9,10]^

Accordingly, this retrospective observational study aimed to examine associations between routinely collected admission nursing assessment indicators and in-hospital adverse outcomes among hospitalized patients with AF. We hypothesized that higher fall risk, poorer functional status, greater nutritional risk, lower serum albumin, and higher pressure-injury risk at admission would be associated with adverse outcomes. The analysis was intended to identify associations and potential complementary markers of vulnerability, not to establish causality or develop a validated prediction model.

## 2. Methods

### 2.1. Study design and setting

This study was approved by the Ethics Committee of Dalian University Affiliated Zhongshan Hospital (2026-DLUZSH-084ks). This retrospective observational study was conducted in a tertiary teaching hospital. Medical records of patients admitted with a diagnosis of atrial fibrillation between January 2021 and December 2024 were reviewed. The diagnosis of atrial fibrillation was confirmed based on electrocardiographic findings documented in the electronic medical record system, including standard 12-lead electrocardiogram or continuous cardiac monitoring, in accordance with established clinical diagnostic criteria. The study was designed to examine the relationship between routinely collected nursing assessment indicators and adverse clinical outcomes during hospitalization. All relevant data were obtained from the hospital’s integrated electronic medical record system and nursing information system.

Patients were eligible for inclusion if they met all of the following criteria – age 18 years or older; hospitalization between January 2021 and December 2024; confirmed diagnosis of atrial fibrillation based on electrocardiographic findings documented in the electronic medical record system, including standard 12-lead electrocardiogram or continuous cardiac monitoring; and availability of complete nursing assessment data within 24 hours after admission, including Morse Fall Scale, Barthel Index, Nutritional Risk Screening 2002, and Braden Scale.

Patients were excluded if they met any of the following criteria – missing key clinical, nursing assessment, or outcome data; discharge or transfer within 24 hours after admission; admission for end-of-life care; or repeated hospitalization during the study period. For patients with multiple hospitalizations during the study period, only the first hospitalization was considered eligible to avoid repeated measurements from the same individual.

The electronic medical record system and nursing information system were searched according to the above criteria. A total of 312 hospitalized patients with atrial fibrillation met the eligibility criteria. No additional patients were excluded after eligibility confirmation. Therefore, the final analytic cohort consisted of 312 patients.

No formal a priori sample size or power calculation was performed because this was a retrospective observational study based on routinely collected electronic medical and nursing records. The sample size was determined by the number of eligible hospitalized patients with atrial fibrillation admitted between January 2021 and December 2024 who met the predefined inclusion and exclusion criteria. A total of 312 patients were included in the final analytic cohort, including 68 patients who experienced adverse outcomes and 244 patients who did not. The number of adverse outcome events was considered when constructing the multivariable logistic regression model, and the number of predictors was restricted to reduce the risk of overfitting.

### 2.2. Study population

Patients were eligible for inclusion if they were 18 years of age or older and had a confirmed diagnosis of atrial fibrillation at the time of hospitalization. Only patients with complete nursing assessment data recorded within the first 24 hours after admission were included to ensure consistency of baseline assessment. Patients were excluded if they had incomplete or missing key clinical, nursing assessment, or outcome data, were discharged or transferred within 24 hours of admission, or were admitted for end-of-life care. For patients with multiple hospitalizations during the study period, only data from the first admission were included to avoid repeated measurements from the same individual. After applying these criteria, 312 eligible patients were included in the final analysis. The electronic medical record and nursing information systems identified 312 patients who met all predefined eligibility criteria. No additional patients were excluded after eligibility confirmation; therefore, the final analytic cohort comprised 312 patients. The selection process is summarized in Figure 1.

**Figure 1.**
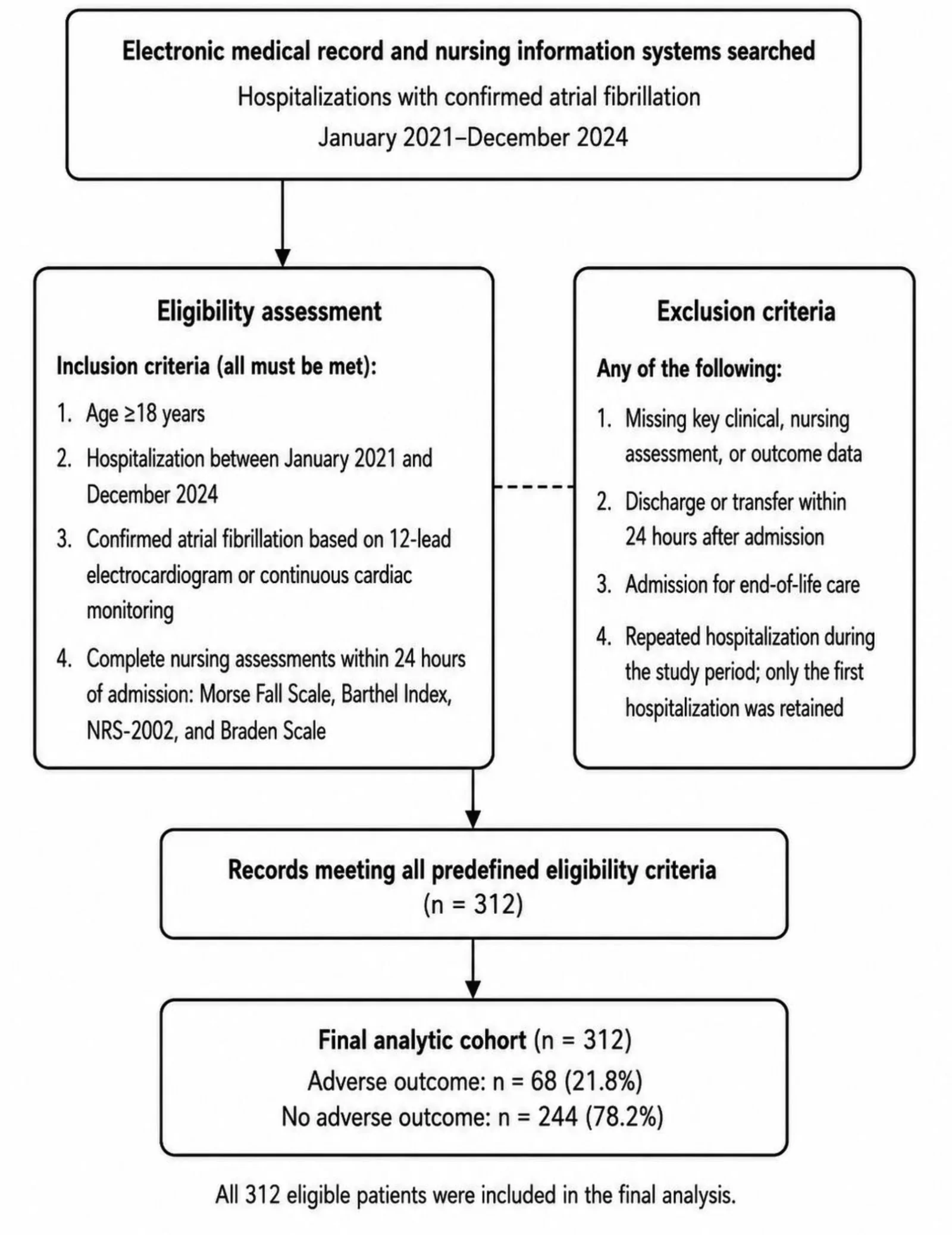
The flow diagram of study.

### 2.3. Definition of adverse outcomes

The primary outcome was a composite in-hospital adverse outcome, defined as the occurrence of one or more clinically significant deterioration events or complications during hospitalization. These events included in-hospital death, unplanned transfer to the intensive care unit, worsening heart failure requiring escalation of treatment, major bleeding requiring medical intervention, or thromboembolic events such as ischemic stroke or systemic embolism. Patients were categorized into the adverse outcome group if they experienced at least one of these events during hospitalization. Patients without any of these events were classified into the non-adverse outcome group. Because the composite outcome includes events of different clinical severity, it was interpreted as an overall indicator of in-hospital clinical deterioration rather than as a single disease-specific endpoint.

### 2.4. Data collection and variables

Demographic and clinical data were extracted from the electronic medical record system by trained investigators using a standardized data extraction protocol. Collected variables included age, sex, body mass index, comorbid conditions, atrial fibrillation type, and length of hospital stay. Comorbidities such as hypertension, heart failure, and chronic kidney disease were identified based on documented clinical diagnoses in the medical records. The type of atrial fibrillation was classified as paroxysmal or persistent according to physician documentation. Risk stratification scores were calculated based on available clinical information. The CHA_2_DS_2_-VASc score was used to assess thromboembolic risk, while the HAS-BLED score was used to estimate bleeding risk. These scores were calculated using standard criteria based on clinical characteristics documented at admission.

### 2.5. Nursing assessment indicators

Nursing assessment data were obtained from the nursing information system. All nursing assessments were performed by registered nurses as part of routine clinical care within 24 hours of admission. Fall risk was evaluated using the Morse Fall Scale, a standardized tool widely used in hospital settings to assess the likelihood of falls. This scale includes items related to fall history, ambulatory status, and mental status, with higher scores indicating greater fall risk. Functional status was assessed using the Barthel Index, which measures a patient’s ability to perform activities of daily living. This index evaluates independence in basic functional tasks, and lower scores indicate greater functional impairment. Nutritional risk was assessed using the Nutritional Risk Screening 2002 (NRS-2002), which considers nutritional status and disease severity. Higher scores indicate greater nutritional risk. Pressure injury risk was evaluated using the Braden Scale, which assesses risk based on sensory perception, mobility, nutrition, and skin condition. Lower scores indicate higher risk of pressure injury. All assessments were performed according to hospital nursing protocols, and assessment results were recorded electronically.

### 2.6. Vital signs and laboratory indicators

Vital signs recorded at admission were obtained from the nursing documentation system. These included heart rate and systolic blood pressure measured during routine clinical evaluation. Laboratory data were obtained from the hospital laboratory information system. Serum albumin level measured within the first 24 hours of admission was included in the analysis as an indicator of nutritional and physiological status.

### 2.7. Statistical analysis

Statistical analysis was performed using SPSS software, version 26.0, IBM Corp., Armonk, NY, USA. Continuous variables were assessed for approximate normality by visual inspection of histograms and Q-Q plots, supplemented by the Shapiro–Wilk test when appropriate. Continuous variables with approximately normal distribution were expressed as mean ± standard deviation and compared using independent-samples t-tests. Continuous variables with markedly skewed distributions were expressed as median and interquartile range and compared using the Mann–Whitney U test. Categorical variables were presented as frequencies and percentages and compared using the chi-square test or Fisher’s exact test, as appropriate. Logistic regression analysis was used to examine associations between clinical variables, nursing assessment indicators, and adverse outcomes. Univariable logistic regression analysis was first performed to explore variables associated with adverse outcomes. Multivariable logistic regression was then performed using variables selected according to both statistical association and clinical relevance. Given that 68 adverse outcome events occurred, the number of variables included in the final model was restricted to reduce the risk of overfitting. Candidate variables included demographic factors, comorbidities, atrial fibrillation type, conventional clinical risk scores, nursing assessment indicators, and serum albumin level. Potential conceptual overlap among nursing indicators was considered during model construction. In particular, the Braden Scale was not retained in the final model because it includes mobility, activity, and nutritional components that overlap conceptually with the Barthel Index and NRS-2002. Atrial fibrillation type was considered during model construction but was not retained in the final model after prioritizing variables most directly aligned with the study hypothesis and nursing assessment framework. The completeness of key clinical variables, nursing assessment indicators, and outcome data was checked before statistical analysis. Because complete nursing assessment data within 24 hours after admission were required for study eligibility, all 312 patients included in the final analytic cohort had complete data for the variables used in the primary analysis. No missing data were identified for the key variables included in the regression analysis. Therefore, no imputation was performed, and no patient was excluded from the final analytic cohort because of missing data.

### 2.8. Ethical considerations

This study was reviewed and approved by the Institutional Review Board of the hospital. Due to the retrospective nature of the study and the use of anonymized data, the requirement for informed consent was waived. All data were handled confidentially, and no identifiable patient information was included in the analysis.

## 3. Results

### 3.1. Baseline demographic and clinical characteristics

A total of 312 eligible hospitalized patients with atrial fibrillation were included in the final analysis after applying the predefined inclusion and exclusion criteria. Among them, 68 patients (21.8%) experienced at least one adverse outcome during hospitalization, while 244 patients (78.2%) did not. The mean age of all patients was 71.4 ± 10.2 years, and 178 patients (57.1%) were male. Patients in the adverse outcome group were older than those in the non-adverse outcome group. Differences were also observed in the prevalence of heart failure, chronic kidney disease, and atrial fibrillation type between groups. CHA2DS2-VASc and HAS-BLED scores were higher in the adverse outcome group, and the length of hospital stay was longer among patients who experienced adverse outcomes (Table 1).

**Table 1:** Baseline demographic and clinical characteristics.

Variables	Total (n = 312)	Adverse outcome (n = 68)	Non-adverse outcome (n = 244)	t/χ^2^	*P*-value
Age (years)	71.4 ± 10.2	74.9 ± 9.6	70.4 ± 10.3	3.12	.002
Male, n (%)	178 (57.1)	36 (52.9)	142 (58.2)	0.61	.435
BMI (kg/m^2^)	24.1 ± 3.8	23.5 ± 3.9	24.3 ± 3.7	1.48	.140
Hypertension, n (%)	201 (64.4)	47 (69.1)	154 (63.1)	0.81	.368
Heart failure, n (%)	103 (33.0)	33 (48.5)	71 (29.1)	8.32	.004
CKD, n (%)	49 (15.7)	16 (23.5)	33 (13.5)	4.08	.043
Persistent AF, n (%)	189 (60.6)	49 (72.1)	140 (57.4)	4.54	.033
CHA_2_DS_2_-VASc score	3.2 ± 1.4	3.8 ± 1.5	3.0 ± 1.3	3.98	<.001
HAS-BLED score	2.1 ± 0.9	2.5 ± 1.0	2.0 ± 0.8	3.89	<.001
Length of stay (days)	9.8 ± 5.4	12.6 ± 6.2	9.0 ± 4.8	4.73	<.001

### 3.2. Nursing assessment indicators

Nursing assessment indicators were evaluated within 24 hours of admission. Differences were observed between the 2 groups in fall risk, functional status, nutritional risk, and pressure injury risk. Patients in the adverse outcome group had higher Morse fall risk scores and nutritional risk scores. Barthel Index scores were lower in this group, reflecting reduced functional independence. Similarly, Braden scores were lower among patients with adverse outcomes. Detailed nursing assessment indicators are shown in Table 2. Because the composite adverse outcome included events of different clinical severity, the individual components of the composite outcome were further summarized separately. The frequency of each component event, including in-hospital death, unplanned ICU transfer, worsening heart failure requiring treatment escalation, major bleeding requiring medical intervention, and thromboembolic events, was reported to improve the clinical interpretability of the composite endpoint (Table S1, Supplemental Digital Content 1).

**Table 2:** Nursing assessment indicators.

Variables	Adverse outcome	Non-adverse outcome	t	*P*-value
Morse fall risk score	45.6 ± 18.2	32.4 ± 15.7	5.48	<.001
Barthel Index score	58.3 ± 21.5	74.6 ± 18.9	−5.66	<.001
NRS score	4.2 ± 1.3	3.1 ± 1.2	5.73	<.001
Braden score	15.9 ± 3.6	17.7 ± 3.2	−3.42	.001

### 3.3. Vital signs and laboratory indicators

Vital signs and selected laboratory indicators at admission were compared between the 2 groups. Heart rate values differed between groups, and systolic blood pressure was lower in patients with adverse outcomes. Albumin levels also differed between the groups. These findings are summarized in Table 3.

**Table 3:** Vital signs and laboratory indicators.

Variables	Adverse outcome	Non-adverse outcome	t	*P*-value
Heart rate (beats/min)	95.2 ± 19.7	86.7 ± 17.9	3.15	.002
Systolic BP (mm Hg)	122.3 ± 18.5	130.2 ± 19.3	2.89	.004
Albumin (g/L)	35.6 ± 4.7	38.2 ± 4.5	3.96	<.001

### 3.4. Univariate logistic regression analysis

Univariate logistic regression analysis was performed to evaluate the association between clinical and nursing assessment variables and adverse outcomes. Several variables, including age, heart failure, fall risk score, functional status score, nutritional risk score, and albumin level, were included in the analysis. The results of the univariate analysis are presented in Table 4.

**Table 4:** Univariate logistic regression analysis.

Variables	OR	95% CI	*P*-value
Age	1.042	1.014–1.071	.003
Heart failure	2.301	1.329–3.985	.003
Fall risk score	1.042	1.025–1.059	<.001
Barthel Index	0.967	0.954–0.981	<.001
NRS score	1.528	1.241–1.883	<.001
Albumin	0.921	0.881–0.962	<.001

### 3.5. Multivariate logistic regression analysis

Multivariable logistic regression analysis was performed to examine adjusted associations between selected clinical and nursing assessment variables and adverse outcomes (Table 5). Candidate variables were selected based on clinical relevance, between-group differences, univariable associations, and consideration of model complexity relative to the number of adverse outcome events.

**Table 5:** Multivariate logistic regression analysis.

Variables	β	OR	95% CI	*P*-value
Fall risk score	0.031	1.031	1.012–1.051	.002
Barthel Index	−0.022	0.978	0.961–0.995	.012
NRS score	0.319	1.376	1.083–1.748	.009
Albumin	−0.048	0.953	0.920–0.988	.010
AF type, persistent vs paroxysmal	0.246	1.279	0.642–2.549	.482
Braden Scale score	−0.043	0.958	0.842–1.090	.516

In the adjusted analysis, Morse Fall Scale score, Barthel Index score, NRS-2002 score, and serum albumin level were independently associated with adverse outcomes. A higher Morse Fall Scale score was associated with increased odds of adverse outcomes (OR = 1.031, 95% CI: 1.012–1.051, *P* = .002). A higher Barthel Index score was associated with lower odds of adverse outcomes (OR = 0.978, 95% CI: 0.961–0.995, *P* = .012). A higher NRS-2002 score was associated with increased odds of adverse outcomes (OR = 1.376, 95% CI: 1.083–1.748, *P* = .009). A higher serum albumin level was associated with lower odds of adverse outcomes (OR = 0.953, 95% CI: 0.920–0.988, *P* = .010).

These findings indicate that nursing assessment indicators reflecting fall risk, functional impairment, nutritional risk, and systemic nutritional status were independently associated with in-hospital adverse outcomes. The regression analysis was not intended to establish a validated clinical prediction model.

## 4. Discussion

In this single-center retrospective cohort, routinely collected admission measures of fall risk, functional dependence, nutritional risk, and serum albumin were independently associated with a heterogeneous composite of in-hospital adverse outcomes among patients with AF. The direction of the associations was internally consistent with a vulnerability phenotype: greater fall and nutritional risk were associated with higher odds of adverse outcomes, whereas greater functional independence and higher serum albumin were associated with lower odds. By contrast, AF type and Braden Scale score did not remain independently associated after adjustment. The findings suggest that nursing-derived measures of functional status, fall risk, nutritional condition, and physiological stability reflect multidimensional vulnerability in this population. Atrial fibrillation commonly affects older adults with multiple comorbidities and reduced physiological reserve. Age-related cardiovascular changes, diminished adaptive capacity, and accumulated disease burden may contribute to increased susceptibility to clinical deterioration during hospitalization.^[11]^ Within this context, nursing assessment indicators provide structured and continuous evaluation of patient status beyond traditional disease-centered parameters. Rather than focusing solely on arrhythmia characteristics or thromboembolic risk, these indicators capture broader aspects of functional and physiological stability that may influence short-term clinical trajectories.^[12,13]^

Functional status, assessed using the Barthel Index, represents an important dimension of patient health. Reduced functional capacity may reflect impaired mobility, decreased muscle strength, and limited physiological resilience. Previous studies have shown that functional impairment is closely associated with poor recovery, prolonged hospitalization, and unfavorable outcomes in hospitalized older adults.^[14,15]^ In patients with atrial fibrillation, functional decline may coexist with hemodynamic instability, heart failure exacerbation, or comorbid chronic conditions, potentially influencing tolerance to acute illness and therapeutic interventions. Functional limitations may also affect medication adherence, mobility during hospitalization, and the ability to participate in rehabilitation activities. Therefore, functional assessment offers clinically meaningful insight into baseline patient vulnerability and may help contextualize disease severity within a broader framework of physical capacity.

Fall risk, evaluated using the Morse Fall Scale, incorporates factors such as ambulation, balance, prior fall history, and cognitive status. Increased fall risk scores may reflect broader mobility limitations and impaired physical coordination. In hospitalized patients, mobility impairment is often linked to deconditioning, sarcopenia, and reduced physiological reserve.^[16]^ For patients with atrial fibrillation, fluctuations in heart rate, episodes of hypotension, or side effects related to rate-control and anticoagulation therapies may further contribute to dizziness or instability. These elements highlight the interaction between cardiovascular dynamics and functional safety. Fall risk assessment therefore extends beyond injury prevention; it may serve as an indirect indicator of overall physiological vulnerability and clinical instability. The association observed in this study suggests that mobility-related assessments may reflect underlying health status that is not fully captured by traditional cardiovascular risk scores.

Nutritional status, measured using the NRS-2002 and serum albumin level, represents another key component of patient evaluation. Malnutrition has been associated with impaired immune response, delayed tissue repair, increased inflammatory burden, and higher complication rates in hospitalized populations.^[17,18]^ In patients with cardiovascular disease, inadequate nutritional status may contribute to reduced cardiac muscle efficiency, impaired wound healing, and diminished tolerance to therapeutic interventions. Serum albumin, although influenced by inflammatory and fluid status, remains a commonly used marker of nutritional and systemic condition. The independent association between nutritional indicators and adverse outcomes observed in this study supports the concept that metabolic and physiological reserves play a meaningful role in clinical stability. Nutritional assessment thus complements cardiovascular evaluation and may help identify patients with limited adaptive capacity during acute illness.

Pressure injury risk, assessed using the Braden Scale, reflects mobility, skin integrity, and nutritional condition.^[19]^ Lower Braden scores may indicate limited movement, compromised tissue perfusion, or systemic frailty. Although pressure injury risk is traditionally viewed as a nursing-sensitive outcome measure, it also represents an integrated assessment of patient immobility and overall physiological status. Reduced mobility and prolonged bed rest may exacerbate deconditioning and increase susceptibility to complications such as infection or thromboembolism. In addition, routinely monitored physiological parameters such as heart rate and blood pressure provide essential information regarding cardiovascular function and hemodynamic stability.^[20]^ Variations in these parameters may signal acute changes in cardiac output or systemic perfusion. Together, nursing assessment indicators and physiological measurements provide a multidimensional evaluation of hospitalized patients with atrial fibrillation, capturing aspects of vulnerability that may not be fully represented by conventional thromboembolic or bleeding risk scores.

The present findings are consistent with recent evidence showing that vulnerability domains beyond conventional AF-specific risk scores are clinically relevant. Yao et al reported in a 2025 systematic review and meta-analysis of 23 studies that frailty was associated with higher risks of all-cause mortality, stroke, and major bleeding in older patients with AF.^[6]^ Similarly, Soler-Espejo et al prospectively studied 1050 patients with AF and found that frailty independently increased the risks of major bleeding and death, whereas moderate-to-severe malnutrition was independently associated with ischemic stroke/transient ischemic attack and mortality.^[7]^ A 2026 systematic review further showed that frailty and advanced age were associated with poorer AF outcomes and greater healthcare burden.^[8]^ In contrast to these studies, which primarily evaluated formal frailty or nutritional constructs and longer-term outcomes, our study focused on nursing assessments already embedded in routine admission workflows and on short-term in-hospital deterioration.

Our findings are also consistent with the broader direction of contemporary AF guidelines. The 2024 ESC guideline emphasizes patient-centered, integrated AF-CARE management and dynamic reassessment, whereas the 2023 ACC/AHA/ACCP/HRS guideline supports comprehensive risk-factor management and cautions against excessive reliance on individual risk scores.^[7,21–24]^ Importantly, the present study does not demonstrate that nursing assessment indicators outperform CHA_2_DS_2_-VASc or HAS-BLED, and incremental discrimination or calibration was not evaluated. Rather, these findings suggest that routinely collected nursing assessments may provide complementary information regarding mobility, functional independence, and nutritional vulnerability that is not directly captured by conventional AF-specific scores.^[25–27]^

## 5. Limitations

Several limitations should be considered. First, the retrospective single-center design precludes causal inference and limits external validity. Second, selection bias is possible because eligibility required complete key clinical, nursing, and outcome data and excluded very short admissions, early transfers, end-of-life admissions, and repeat hospitalizations; the direction and magnitude of this bias cannot be quantified from the available data. Third, information bias may arise from routine electronic documentation, variation in nursing assessments, and potential inter-rater differences. Fourth, residual confounding remains likely because frailty phenotype, cognition, inflammatory markers, medication changes, anticoagulation intensity, socioeconomic factors, and other severity measures were not fully captured. Fifth, the composite outcome combines events with different severity and pathophysiology; although individual components are reported, the study was not powered to model each component separately. Sixth, no a priori sample-size calculation was performed, and 68 events limited model complexity and precision. Seventh, the source analysis did not formally assess linearity of continuous predictors in the logit or conduct sensitivity analyses for alternative functional forms. Eighth, no formal prediction model was developed or validated; discrimination, calibration, internal validation, external validation, and incremental value beyond conventional scores were not assessed. Finally, the patient-flow information available in the supplied dataset begins with 312 records already meeting predefined eligibility criteria, so the number of records screened before eligibility determination cannot be reconstructed without the original screening log.

### 5.1. Knowledge gaps

Important gaps remain. It is unknown whether these nursing measures improve prediction beyond established AF risk scores and routinely available clinical variables; whether their associations are consistent across hospitals, healthcare systems, ethnic groups, and AF treatment strategies; whether changes in nursing scores during hospitalization are more informative than a single admission value; and whether targeted interventions triggered by abnormal nursing assessments can reduce clinically important outcomes. The relative contribution of each component of the composite outcome also remains uncertain.

### 5.2. Future directions

Future research should prospectively enroll larger multicenter AF cohorts with prespecified outcomes, standardized nursing assessment training, and complete reporting of screening and missing data. Analyses should test nonlinear associations, interactions, and component-specific outcomes; evaluate discrimination, calibration, decision-curve utility, and incremental value beyond conventional clinical scores; and use internal and external validation before proposing clinical thresholds. Repeated nursing assessments should be studied as time-varying measures. Ultimately, pragmatic trials are needed to determine whether nursing-assessment-triggered monitoring, mobility support, nutritional intervention, or multidisciplinary care improves patient-centered and cardiovascular outcomes.

## 6. Conclusion

Among 312 hospitalized patients with AF, 68 (21.8%) experienced at least one in-hospital adverse outcome. Higher Morse Fall Scale and NRS-2002 scores were independently associated with higher odds of the composite adverse outcome, whereas higher Barthel Index and serum albumin were independently associated with lower odds. AF type and Braden Scale score were not independently associated after adjustment. These results support routine nursing assessments as complementary markers of patient vulnerability and as potential prompts for closer monitoring, but they do not establish causal effects, validate a prediction tool, or justify changes to guideline-directed AF treatment. Prospective multicenter validation and intervention studies are needed before these measures can influence formal clinical decision pathways.

## Author contributions

**Conceptualization:** Jie Han, Siyu Liu, Nan Li, Ruiqin Zhang.

**Data curation:** Jie Han, Siyu Liu, Nan Li, Ruiqin Zhang.

**Formal analysis:** Jie Han, Siyu Liu, Nan Li, Ruiqin Zhang.

**Funding acquisition:** Ruiqin Zhang.

**Investigation:** Ruiqin Zhang.

**Writing – original draft:** Ruiqin Zhang.

**Writing – review & editing:** Ruiqin Zhang.


