# Supplementary material for: Association between nursing assessment indicators and adverse outcomes in hospitalized patients with atrial fibrillation: A retrospective observational study
Source: Medicine (Baltimore). 2026 Oct 30;105(44):e51239. doi: 10.1097/MD.0000000000051239 (PMC13727202; doi:10.1097/MD.0000000000051239)
Supplement: Supplementary file 1 [file medi-105-e51239-s001.docx]

Supplementary Table 1. Components of the composite adverse outcome

| Component of adverse outcome | Number of patients, n | Percentage of total cohort, % | Percentage among patients with adverse outcomes, % |
| --- | --- | --- | --- |
| In-hospital death | 5 | 1.6 | 7.4 |
| Unplanned ICU transfer | 14 | 4.5 | 20.6 |
| Worsening heart failure requiring treatment escalation | 38 | 12.2 | 55.9 |
| Major bleeding requiring medical intervention | 12 | 3.8 | 17.6 |
| Thromboembolic events | 7 | 2.2 | 10.3 |
| Any adverse outcome | 68 | 21.8 | 100.0 |

Note: The composite adverse outcome was defined as the occurrence of at least one of the above events during hospitalization. Some patients may have experienced more than one component event; therefore, the sum of individual component events may exceed the number of patients with any adverse outcome.
